# Official Proceedings of the Dental Convention, Atlanta, Georgia

**Published:** 1869-09

**Authors:** 


### Proceedings of Societies.

### OFFICIAL PROCEEDINGS OF THE DENTAL CONVENTION, BEGINNING JULY 28TH, IN ATLANTA, GEORGIA.

At the urgent solicitation of several leading members of the Dental profession, Dr. W. H. Morgan, of Nashville, Tenn., issued a call for a Convention at Atlanta, Ga., on the 28th of July, 1869.

In response to this call the following Dentists assembled at the City Hall on the morning of that day, viz:

*Georgia.*—H. Marshall, A. C. Ford, J. D. Thômas, Albert Hape, E. B. Marshall, C. D'Alvigny, Atlanta; H. A. Lowrance, Athens; R A. McDonald, Griffin; J. P. H. Brown, S. G. Holland, Augusta; T. J. Jones, Sparta; H. T. Henry, Covington; J. A. Tigner, Fort Valley; E. W. L'Engle, F. Y. Clark, Savannah; T. W. Hentz, J. Fogle, Columbus; W. H. Burr, Madison; T. J. Crowe, Macon; E. M. Allen, Marietta; B. B. Alford, LaGrange; W. T. Cole, Newnan.

*Alabama.*—S. Rambeau, Montgomery; H. A. McDaniel, C. A. Jordan, Huntsville; H. B. Boyd, Troy; J. G. McAudey, Selma.

*Tennessee.*—W. H. Cook, Cleveland; John Fouché, Knoxville; W. T. Arrington, Memphis; W. H. Morgan, Nashville.

*Kentucky.*—W. G. Redman, Louisville.

*Arkansas*—L. Augspath, Helena.

*South-Carolina.*—W. Reynolds, Columbia; J. M. Day, Aiken.

*Louisiana.*—J. S. Knapp, J. R. Walker; J. G. Angell, G. J. Friedericks, W. S. Chandler, New Orleans.

*Maryland.*—F. J. S. Gorgas, Baltimore.

Professor J. S. Knapp, was called to the Chair, supported by Dr. W. H. Morgan, as Vice-President. Prof. J. F. S. Gorgas, of Baltimore, and Prof. J. G. Angell, of New Orleans, Secretaries.

The Chairman, after stating the objects of the meeting, appointed Drs. Morgan, Gorgas, W. T. Arrington, Chandler, and Jones, a committee to draft a constitution, by-laws, and code of ethics.

During the absence of the Committee, a general expression of views was given relative to the objects of the meeting.

The Committee reported at 11 A. M. The constitution, including by-laws and code of ethics, after being read, was with a few alterations approved and adopted article by article, and as a whole.

At the evening session the following officers were elected:

President—Dr. W. T. Arrington, of Memphis; 1st Vice-President—Dr. Reynolds, of South Carolina; 2d Vice-President—Dr. Augspath, of Arkansas; 3d Vice-President—Dr. McCauley, of Alabama; Corresponding Secretary—Prof. Gorgas, of Maryland; Recording Secretary—Prof. Angell, of Louisiana; Treasurer—Dr. Redman, of Kentucky; Executive Committee—Drs. Morgan, of Tennessee, Knapp, Walker and Chandler, of Louisiana, and Hape, of Georgia.

The retiring Chairman of the Convention, and the President elect of the Association, each in turn, made interesting speeches to the Association on Dental Education and the benefits to be derived from such societies.

The following committees were then appointed by the President:

*On Membership.*—Drs. J. S. Knapp, La.; T. J. Jones, Ga.; G. J. Friedericks, La.

*On Publication.*—Drs. W. S. Chandler, La.; J. R. Walker, La.; J. G. Angell, La.

*Dental Education.*—Drs. F. J. S. Gorgas, Md.; J. P. H. Brown, Ga.; W. M. Reynolds, S. C.

*Physiology and Surgery.*—Drs. F. Y. Clark, Ga.; S. Rambeau, Ala.; J. Fouché, Tenn.

*Dental Chemistry.*—Drs. J. G. McAuley, Ala.; W. H. Burr, Ga.; E. M. Allen, Ga.

*Dental Therapeutics.*—Drs. F. Y. Clark, Ga.; G. J. Friedericks, La.; H. Marshall, Ga.

*Operative Dentistry.*—Drs. W. H. Morgan, Tenn.; J. Fouché, Tenn.; H. A. Lowrance, Ga.

*Mechanical Dentistry.*—Drs. W. G. Redman, Ky.; E. W. L'Engle, Ga.; S. G. Holland, Ga.

*Dental Literature.*—Drs. J. P. H. Brown, Ga.; H. A. McDaniel, Ala.; T. J. Jones, Ga.

*Voluntary Essays.*—Drs. J. R. Walker, La.; J. M. Day, S. C.; W. S. Chandler, La.

*Histology and Microscopy.*—Drs. W. T. Arrington, Tenn.; T. J. Jones, Ga.; John G. Angell, La.

Various interesting letters were read from distinguished members of the Dental profession, breathing a genial and progressive spirit, and expressing most ardent sympathy with the movement, which were placed on file.

Prof. J. S. Knapp, read an exceedingly able paper, written by Prof. A. F. McLain, of New Orleans, on Prophylaxis, or Prevention of Dental Decay—which gave rise to an animated discussion on the same subject.

Dr. J. P. H. Brown read an interesting essay on the progress of Dental science, which, with the paper of Dr. McLain, was referred to the Publication Committee.

Professor Gorgas, of Baltimore, read a paper by Professor S. P. Cutler, of Holly Springs, Miss., entitled Microscopy of the Teeth, for which the thanks of the Asssociation were awarded.

Dr. F. Y. Clark, of Savannah, exhibited a set of artificial teeth, which were worn by General Oglethorpe, and which were curious in respect to their antique style of workmanship.

By invitation, after adjournment of the morning session, the members of the Association visited the Medical College.

At the hour of three, the Association partook of a sumptuous dinner, provided for them by the Council and citizens of Atlanta, at the National Hotel, at which general good feeling prevailed, and many speeches and toasts were given.

At the evening session of the second day, the time was spent almost exclusively in discussions on the treatment of teeth already devitalized, those which have given rise to alveolar abscess and those which have not.

A portion of the members left for their homes on the evening of the second day, but the session of the third and last day was attended by over thirty members, who resumed the discussion of the previous day with much spirit and interest, and extended it so as to embrace especially the subject of preservation of the vitality of exposed pulps.

It is seldom in any Dental Assembly that a subject of this kind is so generally participated in, or so much interest elicited. This may be accounted for by the fact, that its importance is now more generally understood, as well as by the fact that the very able President, Dr. W. T. Arrington, would immediately bring any member to order who wandered from the subject.

A vote of thanks was offered to Mr. Saml. Hape, of the Dental Depot in Atlanta, for providing arrangements for the Association, and for courtesies extended.

A vote of thanks to the City Council of Atlanta, and also to the Railroad companies, and to the Press of Atlanta, for their aid and kind courtesies, were given.

Amid much enthusiasm and high hopes for the future good to be accomplished by the Association, they adjourned to meet in New Orleans, on the 2nd Wednesday in April, 1870.

After the adjournment, committees were formed for the purpose of organizing State Dental Societies in the States of Alabama and Georgia.

